# Sharing Positive Affective States Amongst Rodents

**DOI:** 10.1007/s42761-023-00201-5

**Published:** 2023-08-12

**Authors:** Frédéric Michon, Julian Packheiser, Valeria Gazzola, Christian Keysers

**Affiliations:** 1 Social Brain Lab, Netherlands Institute for Neuroscience, Royal Netherlands Academy of Art and Sciences, Meibergdreef 47, 1105 BA Amsterdam, The Netherlands; 2 Department of Psychology, University of Amsterdam, Amsterdam, The Netherlands

**Keywords:** Reward, Prosociality, Safety, Empathy, Incentive alignment, Cooperation

## Abstract

Group living is thought to benefit from the ability to empathize with others. Much attention has been paid to empathy for the pain of others as an inhibitor of aggression. Empathizing with the positive affect of others has received less attention although it could promote helping by making it vicariously rewarding. Here, we review this latter, nascent literature to show that three components of the ability to empathize with positive emotions are already present in rodents, namely, the ability to perceive, share, and prefer actions that promote positive emotional states of conspecifics. While it has often been argued that empathy evolved as a motivation to care for others, we argue that these tendencies may have selfish benefits that could have stabilized their evolution: approaching others in a positive state can provide information about the source of valuable resources; becoming calmer and optimistic around animals in a calm or positive mood can help adapt to the socially sensed safety level in the environment; and preferring actions also benefiting others can optimize foraging, reduce aggression, and trigger reciprocity. Together, these findings illustrate an emerging field shedding light on the emotional world of rodents and on the biology and evolution of our ability to cooperate in groups.

In humans, group living is thought to benefit from empathizing with others: sharing their pains should motivate against causing such pains, and sharing their joys should reward actions benefiting other group members. Empathy aligns incentives: what is good for you becomes good for me, and what is bad for you becomes bad for me. While empathy was often thought to be uniquely human, a growing number of paradigms have revealed that two of its components already exist in animals: noticing others’ affective states and aligning one’s own affect to that of others. With neuroscientific tools particularly refined in rats and mice, much of this research and this review focus on rodents to understand the biology of these socio-affective components. While, in a related review, we discuss the comparatively large body of evidence showing that rodents notice and share the *distress* of others and avoid actions that *harm* others (Keysers & Gazzola, (n.d.)), here, we highlight nascent evidence for the positive counterpart to these effects: for rodents, the positive affective states of others are detectable, contagious, and reinforcing (see Berthier & Semple, 2018; Reimert et al., 2013; Schwing et al., 2017 for examples in other species).

## Rodents Notice, and Are Attracted by, the Positive Affective States of Others

**Fig. 1:**
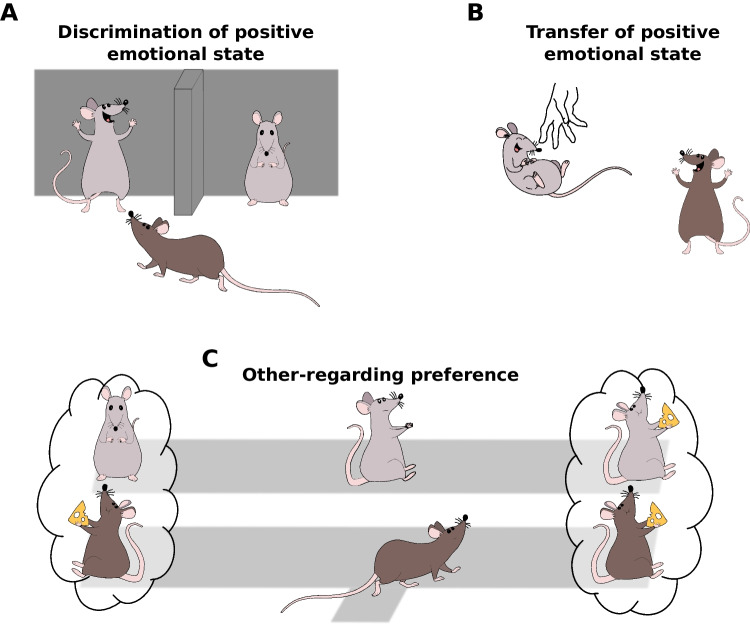
Positive affect transfer paradigms. Paradigms have emerged to study the existence of three ways in which the positive affect of one rodent (gray) influences that of a witness (brown): **A** mice faced with conspecifics in a positive and neutral state spend more time close to the one in the positive affective state. **B** Rats witnessing another being pleasantly tickled show signs of positive affect, and **C** rats prefer arms in a T-maze that provide rewards for both (right) over those providing reward only for the self (left), particularly when the recipient signals their preference

If a mouse is confronted with two other mice, one in a neutral state, the other in a state of relief (triggered by access to water after deprivation), male and female mice spend more time close to the relieved mouse (Ferretti et al., 2019; Scheggia et al., 2020; Fig. 1A). Neuronally, the preference, which appears to depend on olfaction and vision, requires oxytocinergic neurons located in the paraventricular nucleus of the hypothalamus and their projections to the amygdala (Ferretti et al., 2019) and depends on a specific subpopulations of interneurons in the frontal region of the rodent brain (the prelimbic medial prefrontal cortex; Scheggia et al., 2020). As in humans (van der Gaag et al., 2007a, 2007b), similar neural pathways in mice might underly the approach towards a positive and a negative affective state (Ferretti et al., 2019; Scheggia et al., 2020). However, positive affective states seem to be generally attractive to rodents (Ferretti et al., 2019; Scheggia et al., 2020), while negative states might be aversive if too intense or if represented only by odor cues (Ferretti et al., 2019; Rogers-Carter et al., 2018; Scheggia et al., 2020; Valenta & Rigby, 1968).

## Rodents Align Their Affective State to the More Positive State of Others

An animal re-exposed to a footshock-conditioned tone shows reduced fear (e.g., freezing) and stress (e.g., corticosterone) when accompanied by a less stressed bystander (Ishii et al., 2016; Kiyokawa & Takeuchi, 2017; Kiyokawa, Hiroshima et al., 2014; Kiyokawa, Honda et al., 2014)—particularly when that animal is a cagemate (Kiyokawa, Hiroshima et al., 2014; Kiyokawa, Honda et al., 2014), although some buffering occurs also with unfamiliar conspecifics of a related strain (Nakamura et al., 2016). Simply smelling the presence of a conspecific can suffice. Paradigms in which fear levels of two rats are monitored show this transfer to be bidirectional: the more a shocked animal freezes, the more its bystander freezes, but reducing the freezing of this bystander also reduces the freezing of the shocked animal (Cruz et al., 2020; Han et al., 2019). On the neural level, buffering resulted in lower activation of the amygdala, a nucleus located in the medial-temporal lobe (Fuzzo et al., 2015).

Buffering represents an improvement of the affective state of the stressed animal towards the *relatively* more neutral state of the bystander but neither are in truly positive states. In contrast, if humans tickle rats, they emit 50 kHz ultrasonic vocalizations (USVs) and approach the tickling hand as if it were truly rewarding (Panksepp & Burgdorf, 2003). Interestingly, rats observing a rat being tickled (Fig. 1B) also emit 50 kHz USVs and show a playful behavior (Kaufmann et al., 2022), suggesting that this positive state is contagious. The activity of neurons in the somatosensory cortex while observing tickling resembles that while being tickled, suggesting that observers were mirroring (Keysers et al., 2010) the positive tactile sensations of other animals (Kaufmann et al., 2022)—or anticipating similar tickling—akin to neural mirroring of pain in the cingulate (Carrillo et al., 2019).

Others’ positive states can bias cognition. Rats, trained that tone A signals that lever A can provide rewards, while tone B signals that lever B can prevent punishments, are more likely to press lever A when hearing an ambiguous tone, halfway between A and B, when preexposed to a conspecific’s positive vocalizations (Saito et al., 2016).

## Rodents Can Find Witnessing Others Receive Rewards Reinforcing

Both rats and mice prefer actions that provide food for the self and others over actions only feeding the self (Gachomba et al., 2022; Hernandez-Lallement et al., 2015; Márquez et al., 2015; Scheggia et al., 2022; Fig. 1C). This preference depends on the behavior of the recipient: absent if the recipient is a toy (Scheggia et al., 2022) and increased if the recipient signals its preference for the mutually rewarding side (Gachomba et al., 2022; Márquez et al., 2015). It is modulated by hierarchy (Gachomba et al., 2022; Scheggia et al., 2022) and holds even when requiring effort (Scheggia et al., 2022). In rats, it is independent of sex and familiarity (Gachomba et al., 2022). In mice, males invest more effort, and familiarity with the recipient increases the effect (Scheggia et al., 2022). This preference is unlikely to be due to inequity aversion alone: even if the alternative options never deliver rewards to the decision-maker, mice prefer delivering rewards to another mice over delivering no reward at all (Scheggia et al., 2022).

Neuronally, these other-regarding preferences depend on the projections of neurons located in the basolateral part of the amygdala to the prelimbic cortex (Scheggia et al., 2022). The first time, rodents observing others consume a reward triggers a dopamine release in the striatum potentially reinforcing these donating actions (Kashtelyan et al., 2014).

## The Adaptive Value of Sharing the Positive Affective States of Others

What benefit do rodents draw from their ability to perceive, share, and act upon the positive emotional states of others? How does it compare to the human empathic experience? In humans, empathy is used to refer to an “other-regarding,” consciously accessible feeling of what others are feeling, with an awareness that the feeling is on behalf of the other. None of the rodent experiments above yet provide evidence for or against rats or mice *consciously* feeling what others feel, or being *aware* of the source of the shared affective state. Claiming evidence for empathy in rodents may thus be misleading (see Keysers and Gazzola, (n.d.); Kret et al., 2022 for related discussions on the study of feelings in animals). Functionally, some suggest that empathy serves to care for others and evolved to motivate parents to altruistically care for their offsprings (de Waal, 2008; de Waal & Preston, 2017; Preston & Waal, 2002). Below, we invite us to consider a number of more selfish benefits that may explain the evolution of its precursors.

It is adaptive to learn about one’s environment, and if another mouse is in an altered emotional state, it is probably because something happened to it worth learning about. Approaching a conspecific who recently found water or food, for instance, is likely to benefit future foraging by leading to such resources and smelling cues on the lucky rat about the nature and location of what was consumed (Galef, 2012).

It is critical for survival to accurately assess the risks and opportunities of the environment. Using the behavior of others to assess the current risk/safety level improves this balance, as they may have access to information unavailable to the observer (Han et al., 2019)*.* The social buffering, perceptual biases, and transmitted playfulness we describe empower animals’ to adapt to this socially transmitted information. Social information becomes a Bayesian prior that allows an animal to anticipate what may happen to itself—rather than a way to perceive and care about the state of others (Keysers et al., 2022).

Finally, preferring locations that contain food for multiple animals is a valuable heuristic to locate abundant food sources and reduce aggression and competition. That rodents are more generous to those that were generous to them (Engelhardt & Taborsky, 2022; Schweinfurth & Taborsky, 2018; Schweinfurth et al., 2017) makes actions benefiting others ultimately rewarding for the self through reciprocity.

These selfish benefits provide a parsimonious, selfish explanation for the development of a sensitivity to the positive emotions of others. Importantly, they also have undeniable benefits for social living, by allowing groups to exchange information, direct foraging to abundant sources of food benefiting all, reduce competition, and align incentives to promote collaboration.

The degree to which the transfer of positive and negative emotions depends on similar or different neural substrates and is differentially modulated by factors including the species, strain, familiarity, and other genetic and environmental factors is a fascinating question that has so far not been systematically investigated. The transmission of positive affective states seems to produce less robust and replicable effects than negative states. For instance, rats and mice attend vigorously to a conspecific receiving footshocks (Carrillo et al., 2019), but are easily distracted from a conspecific consuming food. This may represent a survival imperative—dangers require more immediate reactions than reinforcers—or the higher novelty of footshocks over the daily experiences of witnessing cagemates eat. Yet, there might be a more fundamental difference. Threats are often bad news for all members of a group, and activating one’s fear when witnessing that of a conspecific helps prepare to react to this shared threat (Keysers & Gazzola, 2021; Keysers et al., 2022). The same is true for social buffering, where safety signals regard all conspecifics, and effects are easily replicated. In contrast, conspecifics are often competitors for food and mates, and witnessing others consume rewards may thus be intrinsically more ambivalent: activating one’s reward pathway is adaptive, to learn about rewarding location and actions; but more negative, anger-like reactions may be necessary to ensure competitive access. Accordingly, finding unambiguous read-out of positive affect in the observer may be more difficult; and factors such as the hierarchy, kinship, familiarity, and history of reciprocity across the animals are likely to influence the balance of reactions and make replications interesting but difficult (see Engelhardt & Taborsky, 2022 and Gachomba et al., 2022 for examples.)

## Additional Information

### Availability of Data and Materials

Data sharing not applicable to this article as no datasets were generated or analyzed for this review paper.
